# Embryotoxicity Caused by DON-Induced Oxidative Stress Mediated by Nrf2/HO-1 Pathway

**DOI:** 10.3390/toxins9060188

**Published:** 2017-06-09

**Authors:** Miao Yu, Liangkai Chen, Zhao Peng, Di Wang, Yadong Song, Hanyin Wang, Ping Yao, Hong Yan, Andreas K. Nüssler, Liegang Liu, Wei Yang

**Affiliations:** 1 Department of Nutrition and Food Hygiene, Hubei Key Laboratory of Food Nutrition and Safety, School of Public Health, Tongji Medical College, Huazhong University of Science and Technology, 13 Hangkong Road, Wuhan 430030, China; yumiao@jiangnan.edu.cn (M.Y.); D201678154@hust.edu.cn (L.C.); 15629089940@163.com (Z.P.); wad1983@126.com (D.W.); song465979919@163.com (Y.S.); hanyin.wang@outlook.com (H.W.); yaoping@mails.tjmu.edu.cn (P.Y.); lgliu@mails.tjmu.edu.cn (L.L.); 2 Ministry of Education Key Lab of Environment and Health, School of Public Health, Tongji Medical College, Huazhong University of Science and Technology, 13 Hangkong Road, Wuhan 430030, China; yanhong@mails.tjmu.edu.cn; 3 Wuxi School of Medicine, Jiangnan University, 1800 Lihu Road, Wuxi 214122, China; 4 Department of Traumatology, BG Trauma center, Eberhard Karls University of Tübingen, Schnarrenbergstr. 95, 72076 Tübingen, Germany; Andreasnuessler@googlemail.com

**Keywords:** skeleton abnormalities, reactive oxygen species, anti-oxidative system, Nrf2 translocation

## Abstract

Deoxynivalenol (DON) belongs to the type B group of trichothecenes family, which is composed of sesquiterpenoid metabolites produced by Fusarium and other fungi in grain. DON may cause various toxicities, such as cytotoxicity, immunotoxicity, genotoxicity as well as teratogenicity and carcinogenicity. In the present study, we focus on a hypothesis that DON alters the expressions of Nrf2/HO-1 pathway by inducing embryotoxicity in C57BL/6 mouse (5.0, 2.5, 1.0, and 0 mg/kg/day) and BeWo cell lines (0 and 50 nM; 3 h, 12 h and 24 h). Our results indicate that DON treatment in mice during pregnancy leads to ROS accumulation in the placenta, which results in embryotoxicity. At the same time Nrf2/HO-1 pathway is up-regulated by ROS to protect placenta cells from oxidative damage. In DON-treated BeWo cells, the level of ROS has time–effect and dose–effect relationships with HO-1 expression. Moderate increase in HO-1 protects the cell from oxidative damage, while excessive increase in HO-1 aggravates the oxidative damage, which is called in some studies the “threshold effect”. Therefore, oxidative stress may be the critical molecular mechanism for DON-induced embryotoxicity. Besides, Nrf2/HO-1 pathway accompanied by the “threshold effect” also plays an important role against DON-induced oxidative damage in this process.

## 1. Introduction

The trichothecenes mycotoxins, often found in food and other organic substrates, are a large group of structurally related sesquiterpenoid metabolites produced by *Fusarium* and other fungi [1,2,3]. Deoxynivalenol (vomitoxin; DON) belongs to the type B group of trichothecenes family and is prevalent worldwide in crops used for food and feed production [4]. Due to its extremely high residual concentration, DON has been considered as one of the most dangerous naturally occurring pollutants [5]. Studies have documented that DON affects animal and human health by causing acute toxicities, such as temporary dizziness, nausea, vomiting, diarrhea, etc. [6,7,8]. Besides, DON also induces cytotoxicity, immunotoxicity, genotoxicity, teratogenicity and carcinogenicity [9,10,11].

Early in 1982, embryotoxicity of DON was first studied in Swiss-Webster mice, but the dose–response relationship was not apparent [12,13]. In the present study, we focus on DON-induced embryonic and developmental toxicities and its possible molecular mechanisms. It is known that embryos during intrauterine growth period are highly sensitive to the exogenous substrates with Gestation Day (GD) 9.5–11.5 being the most sensitive period. This period is also critical for placenta development and the formation of placenta-fetal circulation. As DON can transport across the placental barrier [14], a relatively low-dose maternal DON exposure during pregnancy can result in developmental toxicities for embryos. The main toxic effects are weight restriction, developmental disorders of skeletons and organs [11,15,16,17]. Although some reports on embryotoxicity of DON indicated that oxidative stress can cause various pregnancy-related disorders, such as spontaneous abortions, embryopathies, fetal growth restriction, low birth weight, etc. [18], the related molecular mechanisms in DON-induced embryotoxicity are still unknown.

Excessive accumulation of reactive oxygen species (ROS) in placenta cells affects the intrauterine development of embryos through the placenta-fetal circulation, which could directly or indirectly lead to fetal death, skeleton and organ abnormalities, or to rise in the incidences of obesity, diabetes mellitus and cardiovascular diseases of offspring [19,20,21]. Based on these prominent findings, it has become apparent that oxidative damage of placenta on early stages plays a pivotal role in predicting the fetal development, especially when the developmental toxicities are associated with exposure to exogenous pollutants [22]. The previous study has also shown that DON inhibits the vitality and function of mammal gestational trophocytes, which may affect the growth of the placenta, the formation of the placental barrier and the function of maternal-fetal circulation [14]. As for these pathological results, some studies have also suggested that intracellular ROS accumulation caused by oxidative stress is one of the most important factors of DON-induced early toxicities [23,24]. Therefore, we assume that DON exposure during pregnancy may cause oxidative stress of the placenta, which eventually leads to embryonic developmental toxicities. However, we could not ignore the fact that there is still lack of evidence for a regulated mechanism of DON-induced oxidative damage at present.

A previous study has shown that through interaction with the ribosome DON activates Mitogen-Activated Protein Kinases (MAPKs) pathway and thus mediates a series of downstream molecular effects, including apoptosis, immune response and oxidative stress [25]. At the same time, another study found the bidirectional regulation effect (time and dose dependent) of DON on the MAPKs pathway [26]. Both studies are only preliminary discussions, which urgently require further investigations. MAPKs are series of important signaling components that transfer the extracellular signals into intracellular responses through continuous phosphorylation cascades. When various factors activate the pathway, many transcription factors are phosphorylated, such as NF-E2-related factor 2 (Nrf2), c-*myc*, etc. Nrf2 is combined with Kelch-like ECH-associated protein 1 (Keap-1, the inhibitor of Nrf2) under a quiescent condition in the cytoplasm. The Nrf2-Keap1 signaling pathway is one of the most important cell defense and survival pathways [27]. Once activated, Nrf2 translocates to nucleus, binds specifically to antioxidant response element (ARE), and thus activates the expressions of various enzymes, including heme oxygenase-1 (HO-1), glutathione (GSH), glutathione peroxidase (GPx), etc. [28]. HO-1, a kind of heat shock protein (HSP), is the crucial enzyme in degrading heme to biliverdin, carbon monoxide (CO) and iron. Nrf2-induced HO-1 expression has been considered to be one of the most important intracellular antioxidant mechanisms over the years [29]. Our prior studies have also documented Nrf2/HO-1 as a potential pathway against oxidative damage in animal model [30,31] or in human primary lymphocytes under the DON or quinocetone conditions [10]. At the same time, the protective effect of HO-1 has a threshold [32]. The expression of Nrf2/HO-1 pathway is up-regulated in the early stage of ROS accumulation. Once the ROS accumulation exceeds a certain extent, the expression will be inhibited. We assume that ROS regulates the Nrf2/HO-1 pathway in a “time-dependent” and “dose-dependent” bidirectional pattern. HO-1 may become a new effect target in the process of oxidative damage caused by exogenous substances. 

It is noteworthy that the expression of HO-1 is closely related to the development of the placenta and the formation of placenta-fetal circulation. One study showed that HO-1 protein expression was found in the ectoplacental cone in GD 6.5 and the placenta expressed a high level of HO-1mRNA in GD 13.5–14.5 [33]. Besides, HO-1 deletion in mice has pathological consequences for pregnancy, such as intrauterine fetal growth restriction, fetal lethality, etc. [34]. Although the effect of DON on the expression of HO-1 in placenta and reproductive cell lines (BeWo) is still unknown, studies of T-2 toxin, another mycotoxin of trichothecenes group, have reported its strong inhibitory effect on HO-1 in vivo [35].

Therefore, in the present study, we hypothesize that DON exposure during pregnancy can lead to severe embryonic developmental toxicities, which may associate with oxidative stress of placenta. We also illustrate that DON-induced oxidative stress may be regulated by the Nrf2/HO-1 signaling pathway. Additionally, data from pregnant animals and BeWo cells were combined to evaluate the embryotoxicity of DON and the expressions of the Nrf2/HO-1 pathway during this process.

This study will cast a new light on the molecular mechanism of DON-induced embryonic developmental toxicities, which may provide a fundamental evidence for risk assessment of DON-induced developmental damage.

## 2. Results

### 2.1. Animal Experiment

All mice survived the experimental period until sacrifice (GD 18.5), no significant mortality, illness or clinical signs were observed during the period.

#### 2.1.1. Body Weight and Food Consumption of the Maternal Mice

Body weight and food consumption of the maternal mice was shown in Figure S1. There was no difference in initial body weight among all four groups (Figure S1A). Through the experimental period, body weight in each group was rising steadily. No significant differences in weight change were observed among groups. During the period of DON oral gavages (GD 9.5–11.5), food consumption in DON-treated groups showed the tendency of rising after initial slight falling, and the falling trend in DON-H group lasted the longest time (Figure S1B). Furthermore, food consumption in DON-M and DON-H groups was fluctuating drastically at the later stage of the experiment (but this change was not significant), while food consumption in Control group was steadily increasing.

#### 2.1.2. Embryonic Survival, Growth and Development of the Embryos

In Table S1, results of embryonic survival, growth and development of the embryos were shown. The resorption rate of the embryos in the DON-H group was significantly higher than in the other groups, while no other significant differences were observed when compared all DON-treated groups with the Control. Notably, in most cases, all the embryos of one female mouse in DON-H group were absorbed, so the remaining embryos were not considered representative. Therefore, the results of embryos abnormalities for this group were not shown in the manuscript.

#### 2.1.3. Visceral and Skeleton Morphology of the Embryos

No apparent visceral abnormalities were discovered during the examination. Various skeleton abnormalities of the embryos were displayed in Figure S1 (C–H). By calculating, we found that total malformation rate in the DON-M group (90.9%) was much higher than in the Control and DON-L groups (9.1% and 27.3% respectively, 11 of live embryos were examined for each group). The extremely high total malformation rate in the DON-M group indicated that 2.5 mg/kg/day DON has presented a strong toxicity of embryonic development. Besides, the axial skeleton deformity is the most conspicuous (Figure S1I). 

#### 2.1.4. Pathological Observations in the Placenta of the Maternal Mice 

**Figure 1:**
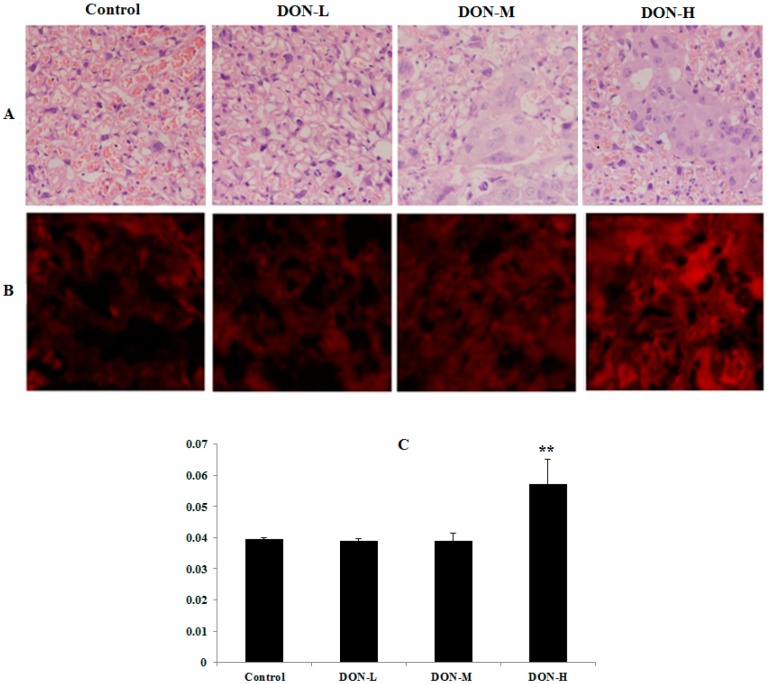
Histopathological changes and ROS detection in the placenta of the maternal mice on GD 18.5: (**A**) fixed placenta sections were stained with hematoxylin and eosin (H&E, 400×); (**B**) dihydroethidium was used to evaluate ROS levels (**B**, 200×); and (**C**) mean fluorescence densities in randomly selected areas of digital images in the placenta were assayed using Image-pro Plus 5.0 (Media Cybernetics, Inc. Rockville, MD, USA) and the values were expressed as means ± SEM (*n* = 3). Significant statistical difference was indicated by ** *p* < 0.01 versus the Control.

In the histopathological examination (Figure 1A, H&E), the structures of the placenta in the Control and DON-L groups were normal. However, for the two other DON-treated groups, granule- and vacuole-like changes were discovered in some placenta syncytiotrophoblast and some trophocytes were necrosis and apoptosis. Furthermore, monocyte invasion and decreased blood supply were observed. 

#### 2.1.5. ROS Levels and Activity of Anti-Oxidative System in the Placenta of the Maternal Mice

The condition of ROS level in the placenta (Figure 1B) shows that DON exposure enhanced the intensities of dihydroethidium probes (red fluorescence) in the placenta of DON-H group. In the DON-L and DON-M groups, there were no remarkable changes compared with the control (Figure 1C). As illustrated in Table 1, the SOD activity and levels of MDA and GSH were measured to determine the activity of anti-oxidative system in the placenta when treated with different doses of DON. The level of HO-1, which plays a crucial role in cellular defense by improving the removal of ROS [36], was also evaluated in this study. Compared with the Control, significant differences in the levels of SOD (DON-L and DON-M), MDA (DON-H) and HO-1 (DON-L) were observed. No significant differences were observed in the GSH level between DON-treated groups compared to the Control.

#### 2.1.6. HO-1, Nrf2 and PIGF Expressions in the Placenta of the Maternal Mice

**Figure 2:**
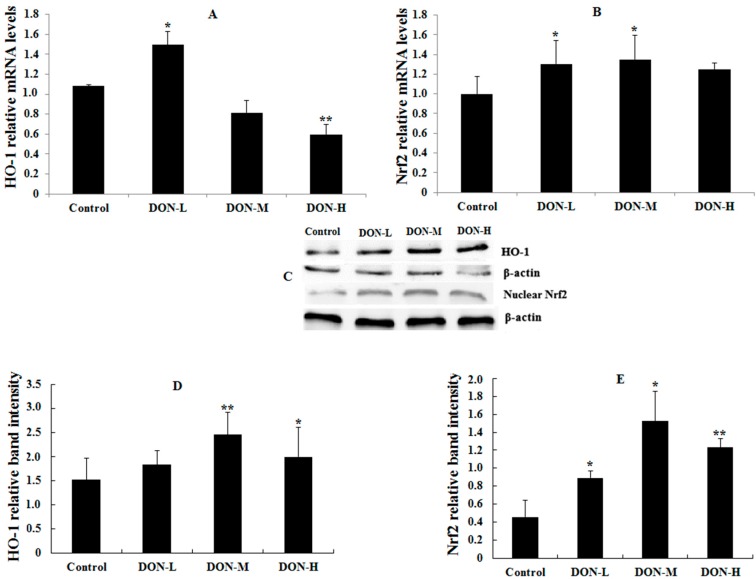
HO-1 and Nrf2 mRNA (**A**,**B**); and protein expressions (**C**–**E**) in the placenta of the maternal mice on GD 18.5. Bars represent the mean ± *SEM* from four independent experiments (*n* = 6). Significant statistical difference was indicated by * *p* < 0.05 and ** *p* < 0.01 versus the Control.

**Figure 3:**
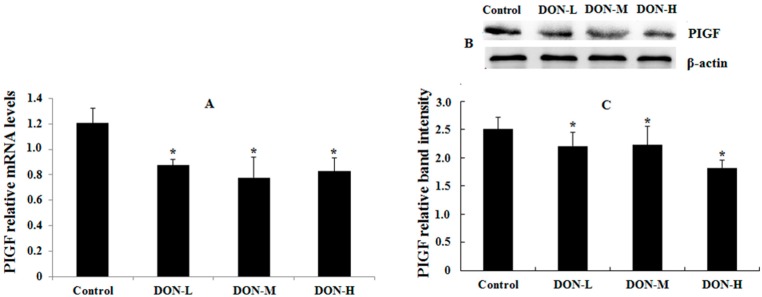
PIGF mRNA (**A**); and protein expressions (**B**,**C**) in the placenta of the maternal mice on GD 18.5. Bars represent the mean ± *SEM* from four independent experiments (*n* = 6). Significant statistical difference was indicated by * *p* < 0.05 versus the Control.

The results of HO-1, Nrf2 and placental growth factor (PIGF, which was used to reflect the maternal condition and intrauterine growth restriction) mRNA and protein expressions were shown in Figure 2 and Figure 3. In general, the mRNA and protein expressions of HO-1 and Nrf2 in DON-treated groups were higher (except for HO-1 mRNA in DON-M and DON-H) and PIGF expressions were significantly lower than in the Control. Besides, the mRNA and protein expressions of HO-1 and Nrf2 showed a downward trend after the initial rise as the dose of DON treatment increased.

#### 2.1.7. Intracellular Translocation of Nrf2 in the Placenta of the Maternal Mice

**Figure 4:**
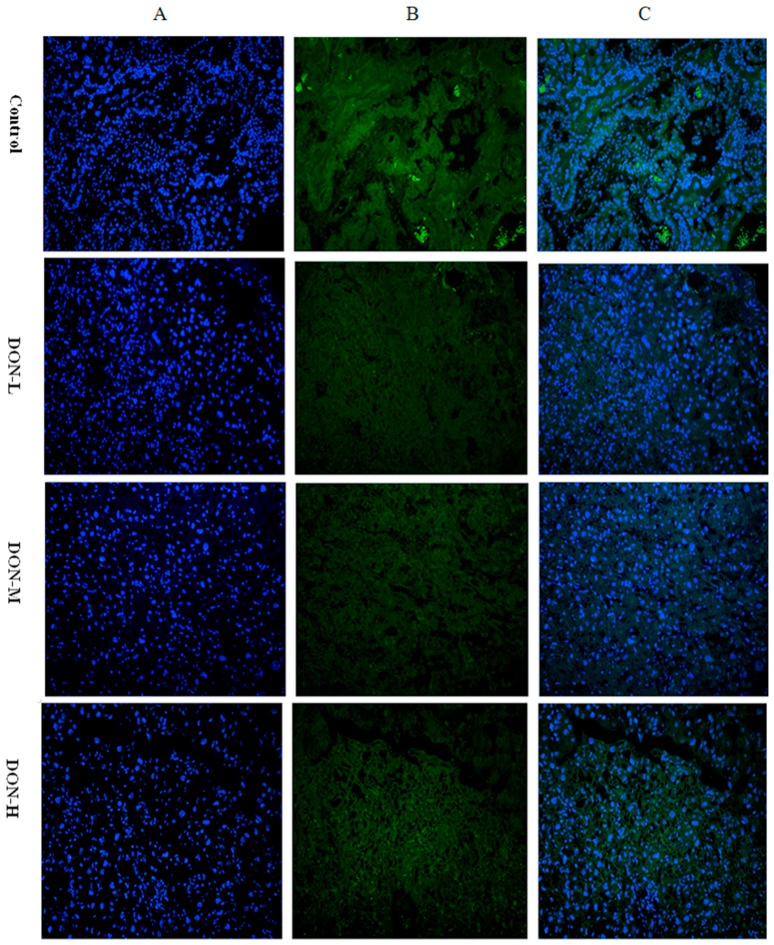

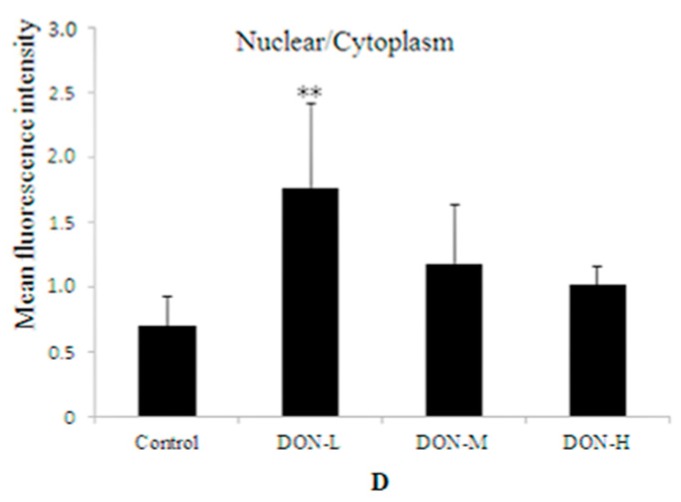
Immunofluorescent pictures of Nrf2 in: nucleus (**A**, 400×); and cytoplasm (**B**) in the placenta of the maternal mice on GD 18.5 (400×); and (**C**, 400×) a overlay of (**A**,**B**). Mean fluorescence densities in randomly selected areas of digital images in the placenta were assayed using Image-pro Plus 5.0 (Media Cybernetics, Inc. Rockville, MD, USA) and the values of nucleus/cytoplasm were expressed as mean ± *SEM* (*n* = 4) in (**D**). Significant statistical difference was indicated by * *p* < 0.05 versus the Control.

Immunofluorescent pictures of Nrf2 in nucleus and cytoplasm are displayed in Figure 4. Mean fluorescence intensity (MFI) of Nrf2 expression in nucleus and cytoplasm were calculated; mean fluorescent intensity of nucleus/cytoplasm in the DON-L group was markedly higher than in the Control, while no significant differences were observed in the two other DON-treated groups.

### 2.2. Cell Experiment

#### 2.2.1. Cell Viability with Different Doses of DON

Cell viability was higher than 90% at 1–12 h, while they decreased to 89.38% (50 nM), 85% (100 nM), 82% (250 nM), 77.75% (500 nM) and 74.56% (750 nM) at 24 h (Figure S2). To explore further details about the impact of lower DON doses of on BeWo cells, we chose 50 nM as the intervention dose. As cell viability remained unchanged at 1–6 h, 3 h was set to reflect the condition of DON-treated BeWo cell as a short period, while 12 h and 24 h as the extended period. Therefore, 3 h, 12 h, and 24 h were chosen as the incubation periods. Besides, the expression of mRNA is extremely sensitive, thus 1 h was added to the PCR test. Hemin and Zinc protoporphyrin (Znpp) were used as agonist and inhibitor of HO-1 respectively.

#### 2.2.2. Indexes of Placental Function in BeWo Cell (the Expression of PIGF)

**Figure 5:**
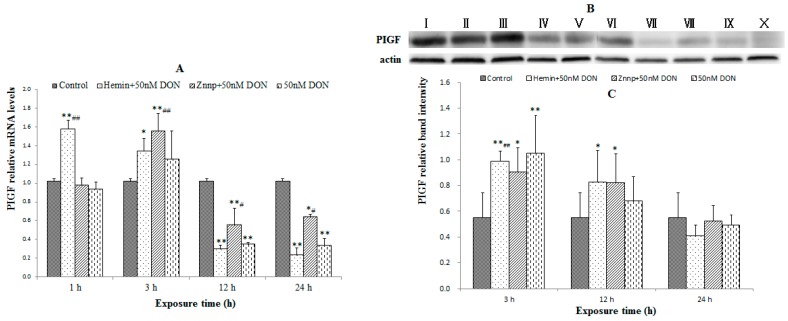
PIGF mRNA (**A**); and protein expressions (**B**,**C**) in BeWo cell treated with different doses of DON. I: Hemin + 50 nM DON, 3 h; II: Znpp + 50 nM DON, 3 h, III: 50 nM DON, 3 h; IV: Hemin + 50 nM DON, 12 h; V: Znpp + 50 nM DON, 12 h; VI: 50 nM DON, 12 h; VII: Hemin + 50 nM DON, 24 h; VIII: Znpp + 50 nM DON, 24 h; IX: 50 nM DON, 24 h; X: Control. Bars represent the mean ± SEM, significant statistical difference was indicated by * *p* < 0.05 and ** *p* < 0.01 versus the Control group, and ^#^ *p* < 0.05 and ^##^ *p* < 0.01 versus the 50 nM DON group with the same time of duration.

In Figure 5A, Hemin increased PIGF mRNA in 1 h (*p* < 0.05) and 3 h (*p* < 0.01), while Znpp improved DON-induced down-regulation of PIGF mRNA in 12 h and 24 h (*p* < 0.05). PIGF protein expression (Figure 5B,C) was similar, but it showed a delayed effect compared to the mRNA expression. This result indicated the inhibited function of BeWo cells induced by DON treatment.

#### 2.2.3. The Level of ROS in BeWo Cell

**Figure 6:**
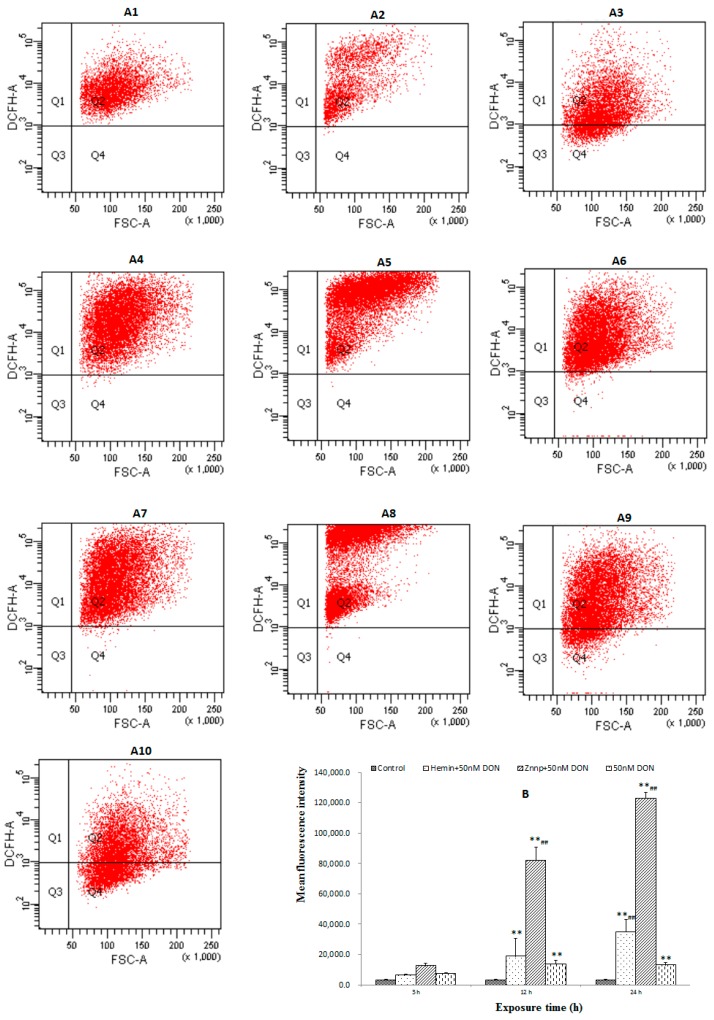
The level of ROS in BeWo cell treated with different doses of DON: (**A**) flow cytometry scatter plot (**A1**: Hemin, 3 h; **A2**: Znpp, 3 h; **A3**: 50 nM DON, 3 h; **A4**: Hemin, 12 h; **A5**: Znpp, 12 h; **A6**: 50 nM DON, 12 h; **A7**: Hemin, 24 h; **A8**: Znpp, 24 h; **A9**: 50 nM DON, 24 h; **A10**: Control); and (**B**) mean fluorescence intensity. Bars represent the mean ± SEM, and significant statistical difference was indicated by ** *p* < 0.01 versus the Control group, and ^##^ *p* < 0.01 versus the 50 nM DON group with the same time of duration.

No significant difference was found among groups (Figure 6) in 3 h. However, Hemin significantly increased ROS level in 24 h (*p* < 0.01) while Znpp significantly increased it in both 12 h and 24 h (*p* < 0.01). It indicated that suppressed HO-1 would lead to excessive accumulation of ROS.

#### 2.2.4. Activity of Anti-Oxidative System in BeWo Cells

The activity of SOD was significantly increased by Hemin in 3 h, but it was significantly decreased in 12 h and 24 h (Table 2). Furthermore, Znpp considerably increased the activity of SOD in 12 h and the level of GSH in 24 h. This result indicated that Hemin up-regulated the antioxidant ability of cell in early stage while Znpp would replace its function soon afterward.

#### 2.2.5. The Expressions of Nrf2 and HO-1 in BeWo Cells

**Figure 7:**
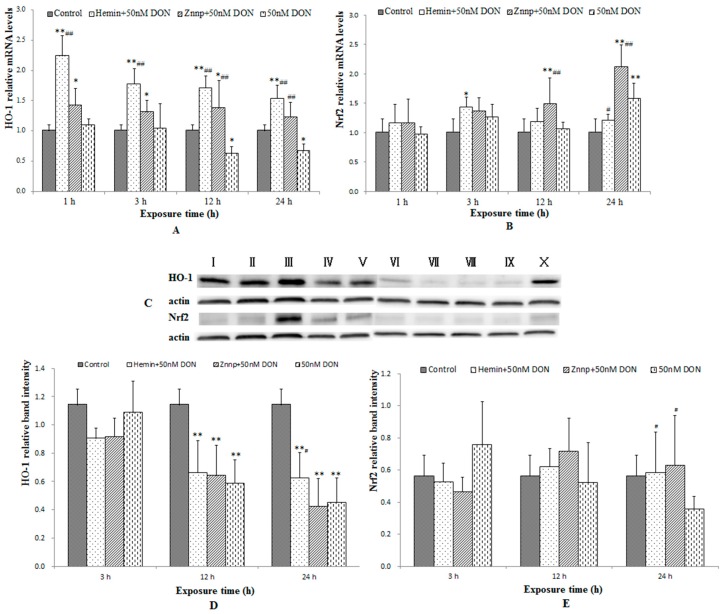
HO-1 and Nrf2 mRNA (**A**,**B**); and protein expressions (**C**–**E**) in BeWo cell treated with different doses of DON. I: Hemin + 50 nM DON, 3 h; II: Znpp + 50 nM DON, 3 h, III: 50 nM DON, 3 h; IV: Hemin + 50 nM DON, 12 h; V: Znpp + 50 nM DON, 12 h; VI: 50 nM DON, 12 h; VII: Hemin + 50 nM DON, 24 h; VIII: Znpp + 50 nM DON, 24 h; IX: 50 nM DON, 24 h; X: Control. Bars represent the mean ± SEM, significant statistical difference was indicated by * *p* < 0.05 and ** *p* < 0.01 versus the Control group, and ^#^ *p* < 0.05 and ^##^ *p* < 0.01 versus the 50 nM DON group with the same time of duration.

The expressions of Nrf2 and HO-1 in BeWo cells were displayed in Figure 7. The level of HO-1 in BeWo cells were significantly increased by Hemin and decreased by Znpp in 3 h. As the intervention time extended, the above functions gradually weakened. The level of HO-1 was higher in the Znpp group than in the Hemin group in 24 h. 

As demonstrated in Figure 7, Hemin and Znpp significantly increased HO-1 mRNA expressions at all time points. Hemin considerably increased Nrf2 mRNA expressions in 3 h (*p* < 0.05) and Znpp in 12 h and 24 h (*p* < 0.01). Besides, DON decreased HO-1 protein expressions at all time points and Hemin improved it only in 24 h. Hemin and Znpp improved DON-induced down-regulations of Nrf2 protein expression in 24 h (*p* < 0.05). In brief, the protein expressions of HO-1 and Nrf2 were not as obvious as the mRNA expression. They were similar with the mRNA expression but with a delay trend.

#### 2.2.6. Intracellular Translocation of Nrf2 in BeWo Cell

**Figure 8:**
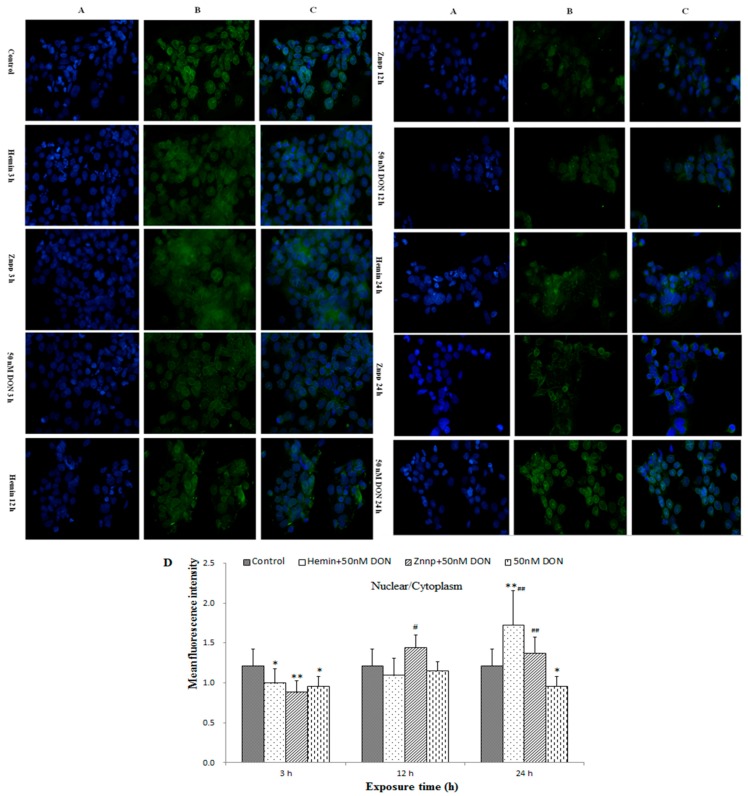
Immunofluorescent pictures of Nrf2 in: nucleus (**A**, 400×); and cytoplasm (**B**, 400×) in BeWo cell treated with different doses of DON; and (**C**, 400×) the overlay of (**A**,**B**). Mean fluorescence densities in randomly selected areas of digital images in the placenta were assayed using Image-pro Plus 5.0 (Media Cybernetics, Inc. Rockville, MD, USA) and the values of nucleus/cytoplasm are expressed as mean ± SEM (*n* = 4) in (**D**). Bars represent the mean ± SEM, and significant statistical difference is indicated by * *p* < 0.05 and ** *p* < 0.01 versus the Control group, and ^#^ *p* < 0.05 and ^#*#*^ *p* < 0.01 versus the 50 nM DON group with the same time of duration.

Immunofluorescent pictures of Nrf2 in BeWo cells are displayed in Figure 8. Fluorescent intensities of nucleus/cytoplasm in the Hemin group (24 h) and Znpp group (12 h and 24 h) were markedly higher than in the Control or 50 nM DON group. Therefore, when exposed to DON for a relatively long time (12 h and 24 h), Nrf2 in BeWo cells pretreated with Hemin and Znpp would translocate from cytoplasm to nucleus.

## 3. Discussion

After GD 9.5–11.5, oral gavages of DON, this mycotoxin exerted its slight feed restriction effect on pregnant mice. However, this effect quickly disappeared and showed no impact on the rise of body weight. The strong fluctuation of food consumption in DON-treated groups (especially DON-M and DON-H) at the later stage of the experiment was probably related to the process of pregnancy. Besides, many factors could influence the food consumption of mice, such as the development of live embryos, dead embryos and resorbed embryos; the formation of deformities; etc. The quite high resorption rate (73.1%) in the DON-H group could explain the declined growth rate of body weight at the later stage of the experiment. The alcian blue and alizarin red double staining revealed that the influence of 2.5 mg/kg/day DON on live embryos was resulted in skeleton abnormalities (no apparent visceral abnormalities in the observation), such as cranial/cervical, rib, clavicle, axial skeleton and limb deformities. These results were in line with the previous literature [12], which demonstrated that some skeletal deformities were present in the 1, 2.5 and 5 mg/kg/day groups, but the dose–response relationship was difficult to ascertain. In conclusion, 2.5 mg/kg/day DON mainly induced skeleton deformities of embryos with the axial skeleton abnormalities while 5.0 mg/kg/day mainly resulted in resorbed embryos. Although previous studies have also reported the DON-induced skeletal deformities [12,16,37], its mechanism is still uncertain. Thus, we elucidated further the possible molecular mechanisms of DON-induced deformities.

We firstly observed relative mechanism of enzymes activities and molecule pathways in placenta tissues. Studies have reported that DNA damage induced by DON in HepG2 cells [24], HT-29 cells [23] and human peripheral blood lymphocytes [10] is probably related to the oxidative stress. It is suggested that toxicity of DON is closely linked to intracellular ROS, and it exerts its toxic effect by a mechanism known as ribotoxic stress response [23]. As the main material of maintaining the stability of internal redox environment, ROS have been proven to play an important role in the regulation of embryonic development [18]. ROS excessive accumulation during pregnancy in the placenta can induce a variety of adverse pregnancy outcomes [19,20,21]. In this study, DON aggravated damage of maternal placenta (mainly in DON-H group), mainly manifesting as granule- and vacuole-like change of syncytiotrophoblast, necrosis and apoptosis of some trophocytes, monocyte invasion and ischemia. Meanwhile, significant ROS generation was discovered in DON-H group. Consequently, it has been indicated that DON treatment during pregnancy led to ROS accumulation in the placenta, which would result in placenta oxidative damage.

Furthermore, MDA is resulted from lipid peroxidation of polyunsaturated fatty acids. Thus, the degree of lipid peroxidation can be estimated by MDA in tissues as a marker for oxidative stress. A rise after decreasing tendency of MDA in DON-treated groups was observed in the experiment. When the exposure dose of DON was low, the antioxidant system of cells was activated. Some antioxidant enzymes in placenta directly reacted with ROS, for instance, the activation of HO-1 was significantly decreased in DON-L group. As we have described before, HO-1 is one of the antioxidant enzymes that protects body against various oxidative damage. Thus, the decreased activation of it indicated its protective effect during DON treatment. As the exposure dose of DON increased, the compensatory ability of antioxidant system appeared to be decreased that eventually caused irreversible lipid peroxidation and oxidative damage. These results are in line with the above literature, which demonstrate that protective effect of HO-1 had a threshold. Besides HO-1, a cell has other antioxidative enzymes (such as SOD, GSH, etc.) to protect it against ROS under normal circumstance. SOD is one of the most important defense mechanisms against toxic effects of oxygen metabolism, while GSH can directly scavenge ROS and its depletion reflects intensive lipid peroxidation [38]. We have further analyzed the reason why their levels remain unchanged. Our suggestion is that the sensitivities of these two indexes for oxidative stress are lower than MDA and HO-1. In addition, DON might not lead to accumulation of singlet oxygen, which can inhibit SOD activities. Altogether, DON treatment during pregnancy leads to ROS generation in the placenta. Although ROS activates the antioxidant system of cells, the decompensation sets in soon afterward that eventually results in remarkable oxidative damage in placenta.

It is well-known that cells use multiple signaling pathways and transcription factors to maintain their balance under stress conditions and specific circumstances. Among these factors, the Nrf2/HO-1 pathway is one of the most important regulators of the cellular stress response [29,39]. In the present study, the expression of Nrf2 and HO-1 showed a downward trend after a slight rise as the dose of DON treatment increased. In detail, the expression of HO-1 mRNA in Control was lower than in DON-L group, but higher than both DON-M and DON-H groups. The immunofluorescent study also found a significantly higher value of Nrf2 expression (nucleus/cytoplasm) in the DON-M group, which was not significant in the DON-H group. These results suggest that the expression of the Nrf2/HO-1 pathway was up-regulated by ROS in the first place, but it was inhibited afterward. 

Our immunofluorescent results indicated the process of Nrf2 translocation from cytoplasm to nucleus. DON treatment induces oxidative stress in cells which leads to dissociation of Nrf2 from Keap-1 and translocation to the nucleus. Then the various antioxidant enzymes (including HO-1, GPx, etc.) increase transcriptions to protect cells from oxidative damage [40]. However, with the increase in the dose of DON the translocation of Nrf2 will be inhibited, and the activity of antioxidant enzymes (e.g., HO-1) will decrease. 

It is noteworthy that PIGF as member of the vascular endothelial growth factor sub-family is a key molecule in angiogenesis and vasculogenesis, in particular during embryogenesis. The main source of PIGF during pregnancy is the placental trophoblast [41]. Therefore, PIGF is used to evaluate the maternal condition and intrauterine growth restriction. The significantly decreased mRNA and protein expressions of PIGF reflected the intrauterine growth restriction of embryos during DON exposure. Therefore, we suppose that the decrease in PIGF expression was related to damage of placental trophoblast which was relevant to DON-induced ROS generation. 

To verify the results in vivo, the cell experiments were conducted combining selective agonist and inhibitor. Hemin and Znpp have been used as HO-1 agonist and inhibitor, respectively, to demonstrate the role of HO-1 in the embryotoxicity induced by DON. In our study, Znpp increased the level of ROS in both 12 h and 24 h. This indicates that suppressed HO-1 leads to excessive accumulation of ROS. Moreover, we found that Hemin up-regulates the antioxidant ability of cell in the early stage, however, this ability was inhibited along with the extending time of DON treatment. This means that though activated HO-1 can increase the antioxidant ability of cell initially, but its continuous activation would decrease it. In other words, although expression of HO-1 is protective against oxidative injury, there may be a level beyond which HO-1 expression becomes detrimental and oxidative injury may occur [42]. This phenomenon could be called as “threshold effect”. 

Similar results have been reported previously. A study suggested that the HO-1 protective action might be restricted to a rather narrow threshold of overexpression, and high levels of HO-1 may even sensitize the cell to oxidative stress through release of reactive iron [43]. In a transient overexpression model, overexpressing HO-1 cDNA levels were no more resistant to oxygen toxicity but some degree of increased oxygen toxicity [44]. Furthermore, when renal BSC-1 cells were exposed to H_2_O_2_ and hemin, a reduction in the viability of the cells was observed. However, long-term exposure (24 h) resulted in the acquisition of some resistance to a further acute challenge of oxidative stress in BSC-1 cells [32]. In vivo, null mutant HO-1^−/−^ fetuses has been studied, the results suggested that HO-1 modulated the release of cellular iron, which may aggravate oxidative injury [45]. Besides, in our present study, DON exacerbated the oxidative stress status that led to the inappropriate expression of HO-1 which reduces the ability of DNA repair in human lymphocytes [10]. Therefore, it is likely that in some circumstances excessive expression of HO-1 may be detrimental.

The reason of the “threshold effect” is that the induction of HO activity may have both pro- and antioxidant sequelae depending on cellular redox potential and the metabolic fate of the heme iron [46]. Specifically, the HO reaction releases iron, which could be involved in deleterious reactions that compete with iron reutilization and sequestration pathways. Under some circumstances, induction of ferritin, a protein that could sequester the redox-active iron released from heme degradation, may counteract iron release. Once ferritin cannot counteract iron release, the HO-1 induction will be detrimental and may aggravate oxidative stress [47].

Finally, we also observed the expressions of the Nrf2/HO-1 pathway in cells. Hemin and Znpp both activated Nrf2 translocation from cytoplasm to nucleus in 12 h and 24 h. Hemin increased Nrf2 and PIGF mRNA expressions at first, while Znpp overtakes its role to improve DON-induced changes in Nrf2 and PIGF mRNA expression in the long run. Therefore, we assume that Hemin exerted a protective effect on BeWo cells in the early stage, but its effect was replaced by Znpp over longer period of time. The reason why the HO-1 mRNA in both Hemin and Znpp groups is up-regulated is that Znpp decreased HO-1 activity while increasing HO-1 expression [48]. It might be explained by the HO-1 mRNA and protein levels increasing over time after decreased HO-1 activity as a rebound or compensation phenomenon [49]. 

In summary, ROS accumulation leads to oxidative stress in BeWo cells. As Nrf2 translocates from the cytoplasm to nucleus, it activates the expression of HO-1 that fulfills the purpose of protecting cells against oxidative damage. At the same time, it should be kept in mind that protective effect of HO-1 against oxidative stress has a “threshold effect”.

## 4. Conclusions

Summarizing the results in vivo and in vitro, embryotoxicity is observed when mice are treated with DON. During the process, excessive accumulation of ROS leads to structural and functional damages of the placenta. Since placenta is the only way for the fetus to get nutrition, its damage causes various adverse pregnancy outcomes, e.g., abortion, stillbirth, or absorption and skeleton abnormalities, which were found in our results. The Nrf2/HO-1 pathway is activated and the expressions of its downstream antioxidant enzymes are increased to protect placenta against oxidative stress. However, with the longer time and higher dose exposure, the antioxidant capacity of cells reaches its limit. Eventually, obvious placental oxidative stress and adverse pregnancy outcomes appear. Our study has for the first time further explored and explained the molecular mechanism of DON-induced embryonic and developmental toxicities. These results may provide basic information for risk monitor and risk assessment.

## 5. Materials and Methods

### 5.1. Chemicals

DON (12, 13-epoxy-3,4,15-trihydroxytrichotec-9-en-8-one, C_15_H_20_O_6_, *M*_W_: 296.32) was purchased from Sigma (St. Louis, MO, USA). The CAS RN is 51481-10-8 (purity ≥ 99%), and the melting point is 151–153 °C. The CCK-8 kit was acquired from the Dojindo chemical company (Kyoto, Japan). The ELISA kit of human HO-1 was acquired from the Cusabio Biotechnology Co., Ltd (Wuhan, China).

### 5.2. Animal Experiment

#### 5.2.1. Animals and Treatment

The study was conducted in accordance with the guidelines for the care and use of laboratory animals (Guide for the Care and Use of Laboratory Animals, National Institute of Health, 1996, Bethesda, MD, USA). Animal experiments described in this study were approved by the Institutional Animal Care and Use Committee at Tongji Medical College, HUST (IACUC number: S407). And date of approval was 28 March 2015. A total of 40 adult female and 20 male C57BL/6 mice were procured from Vital River Laboratory Animal Technology Co. Ltd. (Beijing, China). A Specific Pathogen Free (SPF) room with strictly controlled temperature (23 ± 2 °C), relative humidity (55 ± 5%), light-dark cycle (12–12 h, lights on 7:00–19:00) and ventilation (air-exchange rate of 18 times per hour) was used to feed the animals. The cages and the chip bedding were exchanged twice a week, whereas the feed and tap water were provided ad libitum.

#### 5.2.2. Study Design

After one-week acclimation, 8-week-old female mice were mated with males (two female mice were paired with one male) and Gestation Day 0.5 (GD 0.5) was designated when a vaginal plug was detected. The pregnant mice were randomly divided into 4 groups: DON high-dose group (5.0 mg/kg/day, DON-H), DON medium-dose group (2.5 mg/kg/day, DON-M), DON low-dose group (1.0 mg/kg/day, DON-L) and control group (0 mg/kg/day, Control). The doses used in the present study were referred to the previous studies [12,13,37]. Specifically, DON was dissolved in ultrapure water and diluted to required concentrations with normal saline (the vehicle gavage that control group received). At GD 9.5–11.5, the pregnant mice in each group were given DON by gavage, following the above doses respectively. All the pregnant mice were sacrificed by cervical dislocation at GD 18.5. The placenta was rapidly separated, 3 per litter were placed in 10% neutral buffered formalin and stained with hematoxylin and eosin (H&E), the rest were immediately frozen in liquid nitrogen and stored at −80 °C until analysis. After examining the survival condition and morphology of the embryos collected at GD 18.5, one-third of the fetus in the same litter was fixed in Bouin’s for two weeks for the visceral inspection, the rest were fixed in ethanol. The alcian blue and alizarin red double staining was used to dye the bones Ossified bone was stained red or purple and cartilage stained blue.

#### 5.2.3 ROS in the Maternal Placenta

Dihydroethidium probes were used to reflect the level of ROS in the placenta. Dihydroethidium could cross cell membranes at the presence of ROS and stain nuclei bright red by intercalating with the DNA [50]. At first, the frozen placenta was sliced into 10 μm cross-sections which were then incubated with 5 μmol dihydroethidium (37 °C, 15 min). A Nikon 2000S fluorescence microscope (Nikon, Melville, NY, USA) and the Image-pro Plus 5.0 (Media Cybernetics, Inc., Rockville, MD, USA) were applied respectively to observe and calculate the level of ROS (mean fluorescence intensity per unit area calculated by Image-pro Plus 5.0).

#### 5.2.4. Superoxide Dismutase (SOD), Malondialdehyde (MDA), GSH and HO-1 in the Maternal Placenta

The levels of MDA, GSH, HO-1 and activities of SOD were measured, using commercial assay kits (MDA, GSH, and SOD: Nanjing Jiancheng Institute, Nanjing, China; HO-1: Cusabio, Wuhan, China). The measurements were done according to the instruction of the kits. The specific experimental process is as follows: a Teflon pestle connected to a Braun homogenizer motor was used to homogenize a piece of placenta (specified weight) in ice-cold 50 mM phosphate buffer (pH 7.0, containing 0.1 mM EDTA). Then the 10% (*w*/*v*) tissue homogenate was centrifuged (3500 *g*, 10 min, 4 °C with Eppendorf centrifuge 5804R, Hamburg, Germany) to remove all cell debris and nuclei. Finally, Bicinchoninic Acid assay (Beyotime Biotechnology, Shanghai, China) was used to measure the protein concentrations of the supernatant, which was stored at −20 °C for the subsequent biochemical assays. The results of MDA, GSH, HO-1 levels and activities of SOD are expressed as TBARS nmol/mg protein, mg/g protein, pg/mL and Units/mg protein, respectively.

#### 5.2.5. Real-Time Polymerase Chain Reaction (RT-PCR)

Total RNA of the placenta was isolated using Trizol (Invitrogen, Carlsbad, CA, USA) as per the manufacturer’s instruction of the kit. Then, 2 μg isolated RNA was used to synthesize the complementary DNA at 37 °C for 15 min followed by 85 °C for 5 s (Takara Bio, Dalian, China). RT-PCR was performed with SYBR Premix Ex Taq (Takara Bio, Dalian, China) by an ABI 7900HT real-time thermocycler (Applied Biosystems, Forster, CA, USA). The PCR cycle conditions were 95 °C for 30 s, 40 cycles at 95 °C for 5 s and finally 60 °C for 30 s. Dissociation curves for each gene were obtained to confirm the specificity of the primers. Each sample was analyzed three times and normalized to β-actin. The 2**^−^**^ΔΔCt^ method was used to represent the relative expression of each gene [51]. The primers for the mouse genes were listed as following:

HO-1, Forward: 5′-TGCCCCACTCTACTTCCCTG-3′;
Reverse: 5′-GGCGGTCTTAGCCTCTTCTGT-3′;
Nrf2, Forward: 5′-CCACATTTCCTTCATGGTTTTG-3′;
Reverse: 5′-GACACTTCCAGGGGCACTATCT-3′;
PIGF Forward: 5′-TCCTTCTGAGTCGCTGTAGTGG-3′;
Reverse: 5′-CCTCCTTTCTGCCTTTGTCG-3′;
β-actin, Forward: 5′-AGAGGGAAATCGTGCGTGAC-3′; and
Reverse: 5′-CCATACCCAAGAAGGAAGGCT-3′.

#### 5.2.6. Western Blot Analysis

The extraction of nuclei and cytoplasm protein from maternal placenta was performed based on the manufacturer’s instruction of the kit (P0027, Beyotime Biotechnology, Shanghai, China). The placenta extracts were homogenized with a glass homogenizer in ice-cold lysis buffer (consisting of 50 mM Tris (pH 7.5), 150 mM NaCl, 1% Nonidet P-40, 1% sodium deoxycholate, 1% sodium dodecyl sulfate, 0.1 mM dithiothreitol, 0.05 mM phenylmethylsulfonyl fluoride, 10 mM NaF, 0.5 mM Na_3_VO_4_ and a protease inhibitor cocktail (AMRESCO, Fountain Parkway Solon, OH, USA). The supernatants were collected after incubation at 4 °C for 2 h and centrifugation at 10,000 *g* for 15 min. The protein content was estimated with a Bicinchoninic Acid assay (Beyotime Biotechnology, Shanghai, China). An equal amount of denatured protein from each sample (25 μg per lane) was separated by 12–15% sodium dodecyl sulfate-polyacrylamide gel electrophoresis and then transferred onto a nitrocellulose membrane (BioRad, Hercules, CA, USA). The membranes were blocked with 5% bovine serum albumin (BSA) at room temperature for 1 h and subsequently incubated overnight with specific primary antibodies at 4 °C, followed by incubation with species-specific horseradish peroxidase-conjugated secondary antibodies for 1 h. After washing, immunoreactive bands were detected by either SuperSignal West Pico- or SuperSignal West Dura-enhanced chemiluminescence solution (Pierce, Rockford, IL, USA). Images were captured and analyzed with the GeneSnap (Syngene, Cambridge, UK) and the GeneTools software ( GeneTools 4.0, Syngene, Cambridge, UK), respectively. All data were expressed as ratios to the β-actin loading control. The following primary antibodies were used: anti-Nrf2 (1:50 dilution; sc-722), anti-HO-1 (1:500 dilution; sc-1922-1), anti-PIGF (1:100 dilution; sc-1882) and anti-β-actin (1:200 dilution; sc-47778). The primary antibodies were purchased from Santa Cruz Biotechnology, Santa Cruz, CA, USA.

#### 5.2.7. Immunohistochemistry

Immunohistochemistry and immunofluorescent staining were performed as previously described [52]. In detail, the tissue sections (4 mm) were incubated with Nrf2 antibody, (Sigma, St. Louis, MO, USA), which was diluted in Antibody Diluent Reagent Solution (Invitrogen, Carlsbad, CA, USA) and reactions were carried out overnight at 4 °C. After washing with PBS for three times (5 min each time), the sections were incubated with horse anti-mouse antibody (Sigma, Louis, MO, USA) at room temperature for 30 min. After washing three times with PBS, the slides were stained with 2-(4-amidinophenyl)-6-indolecarbamidine dihydrochloride (DAPI; Beyotime Biotech Inc., Nantong, China) for 2 min to show the locations of nuclei. Images were acquired using an Olympus BX40 microscope (Olympus Corporation, Tokyo, Japan). The Image-pro Plus 5.0 (Media Cybernetics, Inc., Rockville, MD, USA) was applied to calculate the expression of Nrf2.

### 5.3. Cell Experiment

As an immortalized trophoblastic cell line of human origin, BeWo cell has been used in vitro transport model. It was previously demonstrated that DON transports relatively slow across cell monolayer from the maternal side to the fetal side [14]. In this study, BeWo cell was used in vitro to reflect the condition of the placenta during DON exposure with the aim to elucidate the role of placental oxidative stress in embryotoxicity of DON.

BeWo cell was obtained from the American Tissue Culture Collection (ATCC: No. 58311226). BeWo cells were cultured in Dulbecco’s Modified Eagle’s Medium F12 (Gibco, Gibco Inc., Grand Island, NY, USA), which was contained with 10% fetal bovine serum, 1% penicillin/streptomycin (100 U/mL) solution, 2% glutamine and 1% nonessential amino acids (5% CO_2_, 37 °C). Cells were plated on 6-well dishes or 12-well transwell, when reached 80% confluences. After being confluent, 10^4^ cells/well were seeded for the CCK-8 assay (in a 96 well microtiter plate, 4 replicates), grown overnight and then incubated with DON in the appropriate doses and time (0, 50, 100, 250, 500 and 750 nM; 1 h, 3 h, 6 h, 12 h and 24 h). Besides, the cells were first pretreated with the Hemin (10 μM) or Znpp (10 μM), both dissolved in ultrapure water, for 30 min, then washed three times by PBS to remove them. After pretreatment, the cells were treated with different doses of DON for the following experiments. Then, the levels of MDA, GSH, and HO-1, activities of SOD and the gene expressions of HO-1, Nrf2 and PIGF were detected, following the similar testing methods as in the in vivo study. The primers for the human genes were listed as following:

HO-1, Forward: 5′-CAGCATGCCCCAGGATTTG-3′;
Reverse: 5′-AGCTGGATGTTGAGCAGGA-3′; Nrf2,
Forward: 5′-TCCAGTCAGAAACCAGTGGAT-3′;
Reverse: 5′-AATGTCTGCGCCAAAAGCTG-3′; PIGF,
Forward: 5′-TCCCTACTTTGGACAGGAGC-3′;
Reverse: 5′-CTGCAGAAGGAAAGAAGGGG-3′; β-actin,
Forward: 5’-TGACGGGGTCACCCACACTGTGCCCATCTA-3′;
Reverse: 5′-CTAGAAGCATTTGCGGTGGACGATG-3′.

Besides, the level of intracellular ROS was detected using flow cytometric method. Specifically, cells were harvested and centrifuged (3500 *g*, 5 min) at room temperature, then washed with serum-free culture medium and incubated with DCFH-DA (10 μM) for 20 min at 37 °C. After washing three times, the cells were suspended in serum-free culture medium and analyzed for evaluation of the fluorescence intensity by a flow cytometer (BD Accuri C6, Becton, Dickinson and Company, East Rutherford, NJ, USA) (excitation wavelength: 488 nm; emission wavelength: 525 nm).

### 5.4. Statistical Analysis

SPSS 12.0 software package (SPSS Inc., Chicago, IL, USA) was used for statistical analysis. A difference was considered significant when *p* < 0.05 or *p* < 0.01. Data were presented as means ± SEM. If the variances were homogeneous, Analysis of Variance was used. Otherwise, the Kruskal–Wallis non-parametric ANOVA was applied. When statistically significant differences were detected, Dunnett’s multiple tests were employed for establishing comparisons between Control and the other groups.

## Figures and Tables

**Table 1:** Activities of anti-oxidative system in the placenta of the maternal mice on GD 18.5.

Group	SOD	MDA	GSH	HO-1
	(units/mg Protein)	(nmol/mg Protein)	(mg/g Protein)	(pg/mL)
Control	42.71 ± 2.95	3.03 ± 0.29	0.40 ± 0.06	15,167.57 ± 2773.04
DON-L	46.26 ± 1.09 *	2.88 ± 0.77	0.40 ± 0.02	10,246.06 ± 1899.01 *
DON-M	50.88 ± 4.27 *	1.80 ± 0.54	0.40 ± 0.05	11,554.24 ± 4038.46
DON-H	43.05 ± 0.25	5.18 ± 0.48 **	0.37 ± 0.05	13,470.00 ± 1939.74

The values were expressed as means ± *SEM* (*n* = 6). Significant statistical difference was indicated by: * *p* < 0.05 and ** *p* < 0.01 versus the Control.

**Table 2:** Activities of anti-oxidative system in the BeWo cells treated by different doses of DON.

Group	SOD	MDA	GSH	HO-1
	(units/mg Protein)	(nmol/mg Protein)	(μmol/mg Protein)	(ng/mL)
Control	37.79 ± 2.20	3.98 ± 0.29	23.60 ± 2.44	73.33 ± 4.48
Hemin, 3 h	35.18 ± 2.21 ^#^	4.30 ± 0.73 ^#^	11.08 ± 0.30 **^,##^	93.74 ± 2.35 **^,##^
Znpp, 3 h	34.34 ± 2.48	4.18 ± 0.18 ^#^	12.19 ± 1.77 **	52.12 ± 2.59 **^,##^
DON, 3 h	28.58 ± 4.65 *	2.99 ± 1.25	13.37 ± 2.15 **	74.24 ± 5.78
Hemin, 12 h	17.75 ± 6.95 **^,##^	2.50 ± 0.24	4.35 ± 2.44 **	20.71 ± 10.26 **
Znpp, 12 h	46.20 ± 5.17 *^,##^	4.82 ± 0.71	5.17 ± 0.57 **	21.31 ± 3.24 **
DON, 12 h	30.38 ± 3.26 *	3.79 ± 0.39	3.51 ± 0.28 **	19.90 ± 2.88 **
Hemin, 24 h	9.13 ± 6.58 **^,#^	3.97 ± 0.46	2.36 ± 1.74 **	14.55 ± 0.00 **^,#^
Znpp, 24 h	17.16 ± 3.12 **	4.03 ± 0.24	4.72 ± 1.32 **^,##^	21.21 ± 6.00 **^,##^
DON, 24 h	20.22 ± 5.36 **	4.28 ± 0.81	0.16 ± 0.27 **	7.68 ± 1.52 **

The values were expressed as mean ± SEM (*n* = 4). Significant statistical difference was indicated by: * *p* < 0.05 and ** *p* < 0.01 versus the Control group, and ^#^ *p* < 0.05 and ^##^ *p* < 0.01 versus the 50 nM DON group with the same time of duration.

## Supplementary Materials

The following are available online at www.mdpi.com/2072-6651/9/6/188/s1, Figure S1: Mean body weight (A) and food consumption (B) of pregnant mice in different groups. Skeleton abnormalities of the embryos on GD18.5 d (C-H). (C) Normal development of full-body skeletons. D-H showed abnormalities of different regions of the embryos body. (D) Cranial and cervical skeletons. (E) Upper limbs and ribs. (F) Axial skeletons. (G) Lower limbs. (H) Tail. D1, E1, F1, G1 and H1 were the normal control of different regions of the embryos body. (I) shows the percentage of total skeleton abnormalities (11 of live embryos were examined for each group). Blank indicates 0% incidence, Figure S2: The viability of BeWo cell treated with different doses of DON with different time periods. Significant statistical difference was indicated by ** *p* < 0.01 versus the Control, Table S1: Effects of oral administration of DON for GD9.5~11.5 d on GD18.5 day maternal mice placenta and embryos.

## Abbreviations

ARE	antioxidant response element
DON	Deoxynivalenol
DON-H, DON	high dose group
DON-M, DON	medium dose group;
DON-L, DON	low dose group
GD	gestation day
GSH	glutathione
GPx	glutathione peroxidase
HSP	heat shock protein
H&E	hematoxylin and eosin
HO-1	heme oxygenase-1
H_2_O_2_	hydrogen peroxide
•OH	hydroxyl free radical
Keap-1	the inhibitor of Nrf2, Kelch-like ECH-associated protein 1
MDA	Malondialdehyde
MFI	Mean fluorescence intensity
MAPKs	Mitogen-Activated Protein Kinases
Nrf2	NF-E2-related factor 2
NO	nitric oxide
ROS	reactive oxygen species
SPF	Specific Pathogen Free
SOD	Superoxide Dismutase
Znpp	Zinc protoporphyrin
