# Supplementary material for: Embryotoxicity Caused by DON-Induced Oxidative Stress Mediated by Nrf2/HO-1 Pathway
Source: Toxins (Basel). 2017 Jun 9;9(6):188. doi: 10.3390/toxins9060188 (PMC5488038; doi:10.3390/toxins9060188)
Supplement: Supplementary file 1 [file toxins-09-00188-s001.pdf]

Supplementary Materials: Embryotoxicity Caused by DON-Induced Oxidative Stress Mediated by Nrf2/HO-1 Pathway

Miao Yu, Liangkai Chen, Zhao Peng, Di Wang, Yadong Song, Hanying Wang, Ping Yao, Hong Yan, Andreas K. Nüssler, Liegang Liu and Wei Yang

Table S1. Effects of oral administration of DON for GD9.5~11.5 d on GD18.5 day maternal mice placenta and embryos.

Group	Live embryos (%)	Dead embryos (%)	Resorbed embryos (%)
Control	95.0 (38/40)	0 (0/40)	5.0 (2/40)
DON-L	95.7 (44/46)	0 (0/46)	4.3 (2/46)
DON-M	92.8 (39/42)	2.4 (1/42)	4.8 (2/42)
DON-H	26.9 (14/52) **	0 (0/52)	73.1 (38/52) **

Significant statistical difference was indicated by: ***P*< 0.01 versus the Control, *n* = 6 (6 maternal mice were examined in each group).

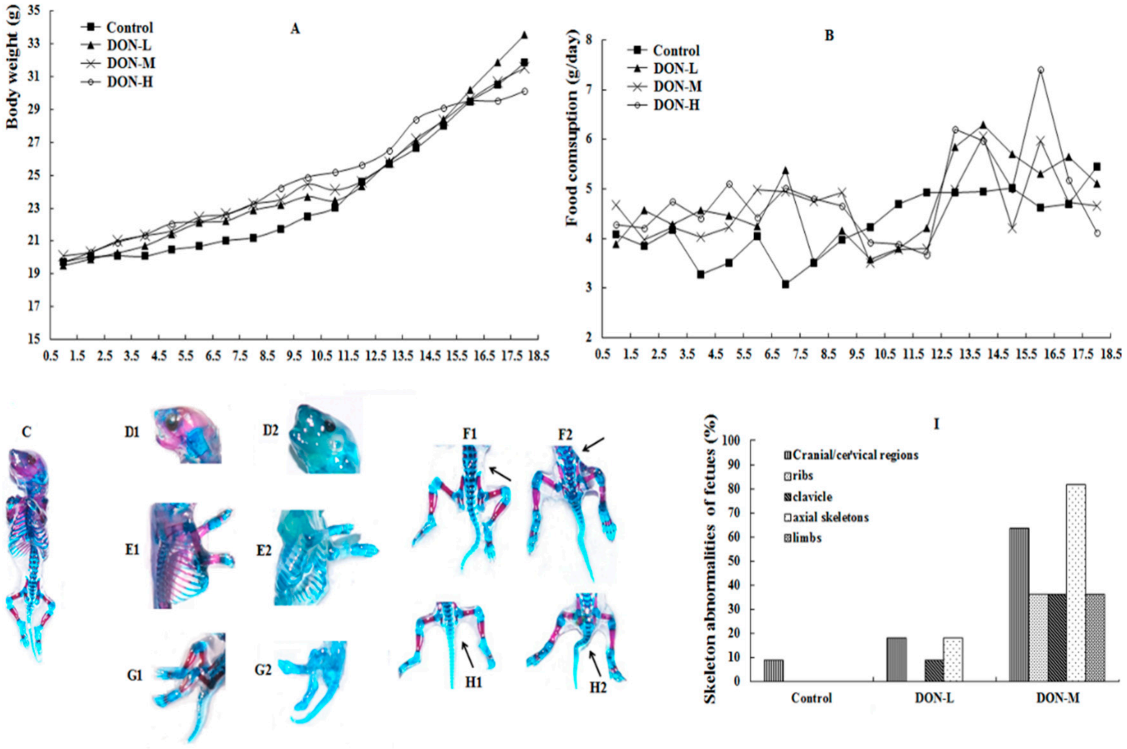

Figure S1. Mean body weight (A) and food consumption (B) of pregnant mice in different groups. Skeleton abnormalities of the embryos on GD18.5 d (C-H). (C) Normal development of full-body skeletons. D-H showed abnormalities of different regions of the embryos body. (D) Cranial and cervical skeletons. (E) Upper limbs and ribs. (F) Axial skeletons. (G) Lower limbs. (H) Tail. D1, E1, F1, G1 and H1 were the normal control of different regions of the embryos body. (I) shows the percentage of total skeleton abnormalities (11 of live embryos were examined for each group). Blank indicates 0% incidence.

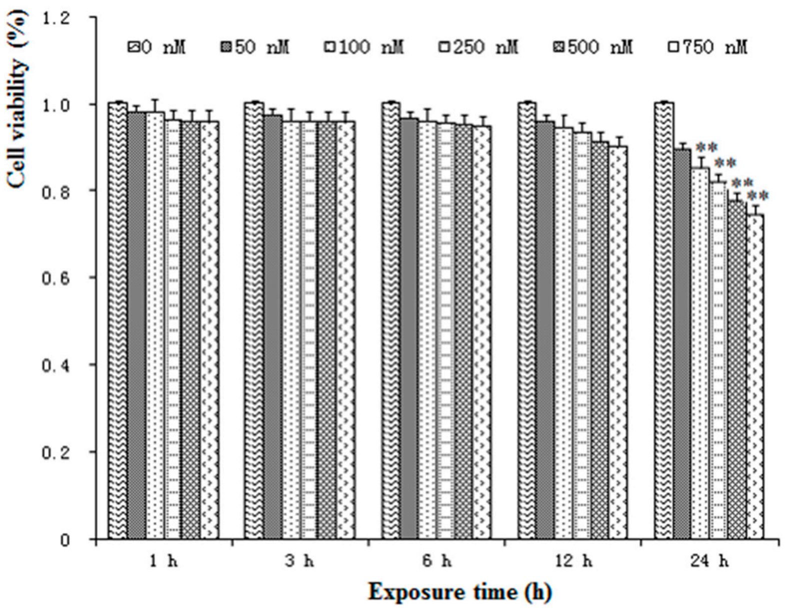

Figure S2. The viability of BeWo cell treated with different doses of DON with different time periods. Significant statistical difference was indicated by $^{**}P < 0.01$ versus the Control.
